# Explosion fatalities in Sweden, 2000–2018

**DOI:** 10.1177/00258024211025228

**Published:** 2021-06-22

**Authors:** Mensura Junuzovic

**Affiliations:** Umeå University, Sweden

**Keywords:** Explosion, blast, death, accident, suicide, homicide

## Abstract

Epidemiological aspects of explosion-related deaths in a civilian setting may bring comprehensive knowledge that is important for prevention efforts. This Swedish national study aimed to describe the extent of such deaths, circumstances and fatal injuries. Data, including all explosion-related deaths in Sweden from 2000 through 2018, were retrieved from the register of the National Board of Forensic Medicine. Among all 87 cases found, accidental deaths accounted for 62%, suicides for 21%, homicides for 7% and undetermined manner of death for the remaining 10% of cases. Most victims died on site. Adult males dominated in the study material, but explosions also killed four children. Explosives were most commonly involved in occupational blast deaths, suicides and homicides, followed by flammable gases and fluids. The incidence showed a significant decrease since the 1980s, based on the incidence rate from this study and a previous Swedish study (1979–1984). As already rare occurrences, blast-related deaths are challenging to prevent. Prevention efforts are needed to restrict the availability of explosives and focus on lowering the occupational risk for injury. In addition, child deaths must not be neglected. A vision of no fatalities is an appropriate goal for acting against explosion-related deaths in a civilian setting.

## Introduction

Explosion-related injury and death are often associated with warfare but may occur also during peacetime. The blast injury panorama in a civil society under peace conditions varies depending on the explosion process (e.g. mechanical or chemical). An explosion always carries a potential risk of injury to those who are close to the blast due to the rapid and sudden release of energy and the pressure wave created. If non-fatal, the incident often leads to physical and mental harm and sequelae to the injured person. Special medical skills necessary for assessment, evaluation and treatment of explosion injuries represent a challenge for emergency care, especially considering that few doctors have experience in the field.^1^

Controlled and planned explosions, which are used in certain professions, require special safety measures and should therefore rarely cause injury and/or death. However, uncontrolled and unplanned explosions may occur due to, e.g. technical errors or as illegal explosions, such as terrorist acts and other criminal activities. Previous studies of blast injuries have focused mainly on a specific type of explosion, e.g. gas explosions,^2,3^ tyre explosions,^4^ pyrotechnic explosions^5^ and explosions in war and terrorism.^6–10^ National studies including all types of explosions are scarce.^11,12^

Epidemiological investigations with long-term trends concerning fatal explosions are lacking in Europe and in other continents. The present study fills this gap and represents an exploratory epidemiological analysis of the incidence of blast fatalities, demographic factors, circumstances and types of injuries.

## Materials and methods

All cases of explosion-related deaths in Sweden in the period from 2000 to 2018 were retrieved from the database of the National Board of Forensic Medicine. A database search for explosion-related fatalities resulted in 87 cases, which forms the sample for this study.

In Sweden, a medico-legal autopsy is performed in recognised and suspected non-natural deaths, such as explosion-related deaths. The National Board of Forensic Medicine is responsible for all such medico-legal investigations. The register contains information about the cause and manner of death (MOD), toxicological findings and information from the police reports. Available variables collected from the register and relevant for the study aim were: age, sex, place and date of death, circumstances, cause and MOD, fatal injuries, and toxicological findings. Illicit drugs were defined according to Swedish law.^13^ In the present study, toxicological findings of alcohol refer to ethanol.

Following data retrieval, cases of undetermined MOD (*n* = 18) were re-evaluated, based on the information from the register. Nine cases were left undetermined, while four cases were reclassified as homicides, one as a suicide and four as accidental deaths.

Statistical methods used were regression analysis and chi-square test (χ^2^). Regression analysis was used to estimate the relationship between mortality incidence and time (years); one analysis regarded the incidence rates in the present study period only; the other analysis also included long-term incidence rates of explosion-related deaths from 1979 through 1984, available from a previous Swedish study.^11^ This long-term analysis included the only previous Swedish study in this issue,^11^ which had also the same sample source (the National Board of Forensic Medicine).

Comparison between the share of alcohol positive and alcohol negative victims in suicides and accidental deaths was done using the χ^2^ test. The statistical analyses were performed using SPSS (version 26) for Windows and statistical significance was defined as a *p*-value < 0.05.

According to the Swedish Ethical Review Act (SFS 2003:460) ethical approval is not required for this study, as the data are not related to living persons but to register data of decedents. The study was approved by Umeå University, Department of Community Medicine and Rehabilitation, Forensic Medicine (13 June 2019) and the data retrieval was approved by the National Board of Forensic Medicine (Dnr X19–90291).

## Results

**Figure 1.**
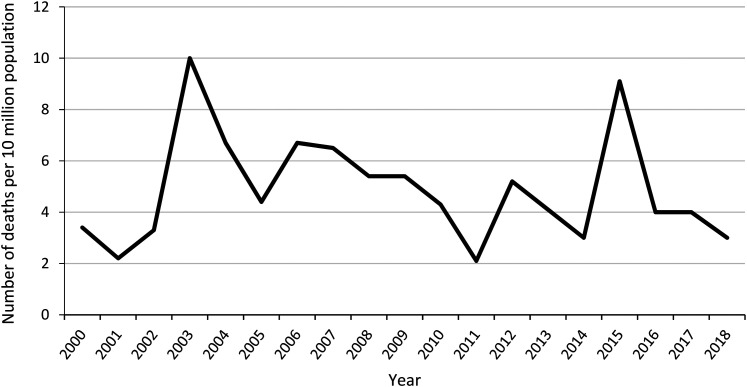
Annual incidence of explosion-related deaths per 10 million population in Sweden 2000–2018.

The annual incidence of explosion-related deaths was 4.9 cases per 10 million population (mean Swedish population during the study period = 9.4 million) (Figure 1). The incidence decreased significantly over time when both the incidence in 1979–1984^11^ and the incidence in the present study period were included in the regression model (*B* coefficient = −0.73, *R*^2^ = 0.53 and *p* < 0.0005). The incidence decreased also when the regression model was applied exclusively on the present study period (2000 through 2018) (*B* coefficient =  −0.08, *R*^2^ = 0.008), but this decrease was not statistically significant (*p* = 0.721).

**Figure 2.**
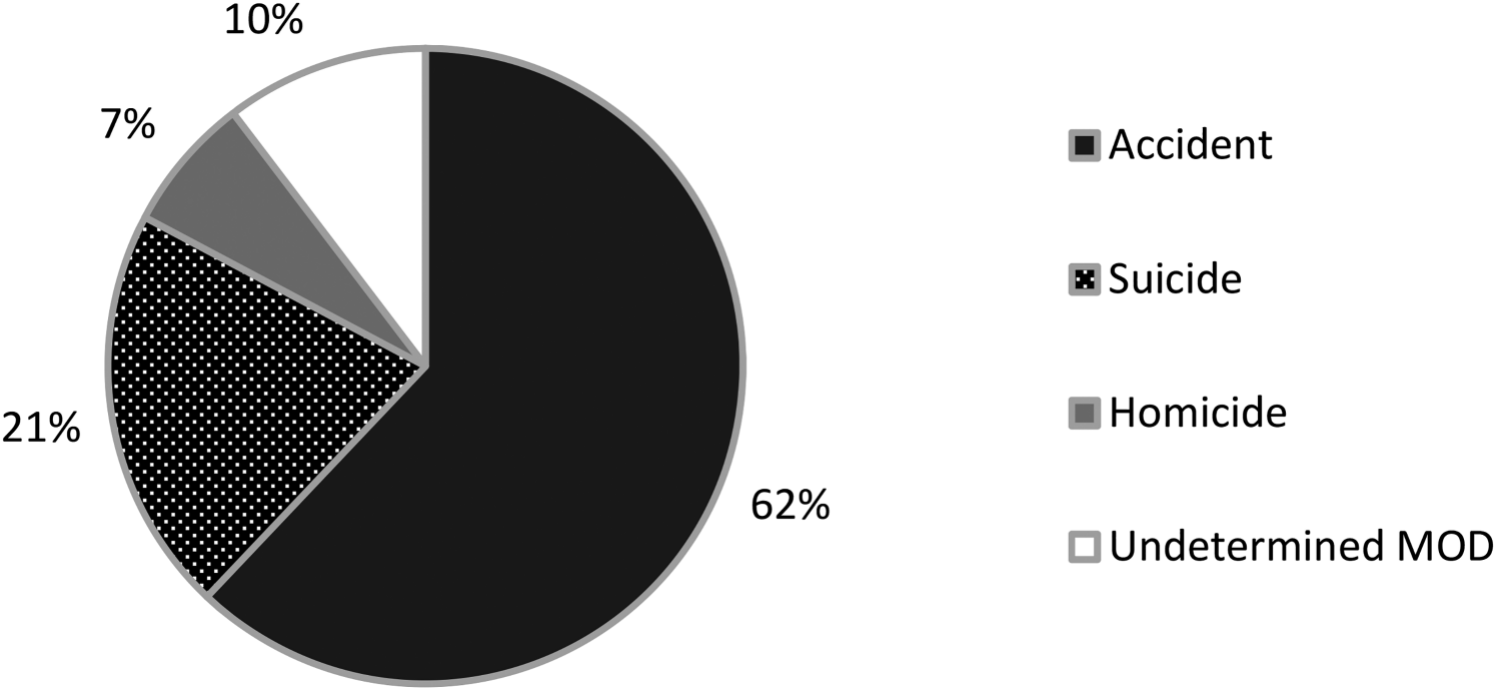
Explosion-related deaths in Sweden 2000–2018, by the manner of death (MOD).

The majority of fatalities were accidental (*n* = 54; 62%), followed by suicides (*n* = 18; 21%), homicides (*n* = 6; 7%) and undetermined MOD (*n* = 9; 10%) (Figure 2). There were 53 accidental events in total; 52 persons were killed in 52 accidental events and two persons were killed in one accidental event. Three homicidal events resulted in six fatalities; four victims were killed in one of these events. Eighteen suicide victims were killed in 18 suicidal events.

Male victims accounted for 91% (79/87) of the study material. Male predominance was found across all MOD categories and males were represented in 94% (51/54) of the accidental deaths, 94% of the suicides (17/18), 83% of the homicides (5/6) and 67% of the cases with undetermined MOD (6/9).

The mean age of victims in accidental events was 46 years (median 51 years, *SD* 18.7), thus lower than the mean of 52 years found in suicides (median 51 years, *SD* 19.9) and 51 years (median 52 years, *SD* 23.5) in cases with undetermined MOD. The lowest mean age of 22 years was found among homicides (median 27 years, *SD* 13.4). Children, all under 10 years, were victims in one accident, in two homicides and in one case of undetermined MOD.

Almost one-third (30%) of the accidental deaths were related to explosions with fires causing quaternary effects such as burns and gas intoxications (Table 1).

**Table 1.** Explosion-related deaths in Sweden 2000–2018 by manner and cause of death, number of fatalities, main injury, blast injury type and survivability.

MOD, type of explosion (ICD-10) (*n* = number of fatalities)	Main blast injuries	Blast injury type	Death at the scene number of fatalities/total (%)
Accidental (*n* = 54)			34/54 (63%)
Explosion and rupture of a gas cylinder (W36) (*n* = 4)	Skull fractures with brain contusion	Secondary and quaternary	2
	Heart and lung lacerations burns		
Explosion and rupture of pressurised tyre, pipe or hose (W37) (*n* = 2)	Electrical injury	Quaternary	1
	Burns		
Explosion and rupture of other specified pressurised devices (W38) (*n* = 8)	Skull fractures with brain contusion	Secondary	7
	Heart and lung lacerations		
Discharge of firework (W39) (*n* = 5)	Skull fractures with brain contusion	Secondary	4
	Heart and lung lacerations		
Explosion of other materials (W40) (*n* = 19)	Multiple injuries of inner organs	Secondary	12
	Skull fractures with brain contusion		
	Heart and lung lacerations		
	Lower extremity vessel injury		
Accidental poisoning by and exposure to other gases and vapours (X47) (*n* = 4)	CO poisoning	Quaternary	3
	Cyanide poisoning		
Burn of the unspecified body region, unspecified degree (T30.0) (*n* = 11)	Burns	Quaternary	5
Exposure to uncontrolled fire, not in building or structure (X01) (*n* = 1)	Toxic inhalational lung injury	Quaternary	0
Suicidal (*n* = 18)			16/18 (89%)
Intentional self-harm by explosive material (X75)	Total bodily laceration	Secondary and quaternary	
	Multiple injuries of inner organs		
	Skull fractures with brain contusion		
	Heart and lung lacerations		
	CO poisoning		
	Burns		
Homicidal (*n* = 6)			4/6 (67%)
Assault by explosive material (X96)	Skull fractures with brain contusion	Secondary	
	Heart and lung lacerations		
Undetermined MOD (*n* = 9)			8/9 (89%)
Contact with explosive material, undetermined intent (Y25)	Skull fractures with brain contusion	Secondary and quaternary	
	CO poisoning		
	Burns		

MOD: manner of death; ICD-10: International Statistical Classification of Diseases and Related Health Problems, 10th Revision; CO: carbon monoxide.

Accidental events occurred both indoors (*n* = 22) and outdoors (*n* = 21), while four occurred in a car and six in an unknown location. More than half of the victims died at the scene and the remaining victims died in hospital (Table 1).

Occupational accidental deaths accounted for 32% (17/54) of all unintentional fatalities. Seven victims were killed in work with explosives. Flammable gases and fluids were involved in six cases during reparation work, and in another four cases with other occupational circumstances. In four fatalities the work included welding.

The majority of suicides (89%) died at the scene from various types of injuries, burns and intoxication (Table 1). Explosives were involved in 13 cases. Most of the cases occurred in a car (*n* = 6) and indoors (*n* = 6), and a suicide note was found in 28% of cases.

In homicides, 67% of the victims died at the scene (Table 1). Explosives were involved in all cases. Four of the cases occurred in a car, one outdoors and one indoors.

All cases of undetermined MOD occurred in explosions with fire; eight indoors and one in a car.

More than half (*n* = 49; 56%) of all victims tested positive for alcohol and/or drugs (Table 2). Positive findings predominated in cases of undetermined MOD, followed by suicides, homicides and accidental deaths. The finding of illicit drugs (mostly cannabis, amphetamine and cocaine) was uncommon (*n* = 9/87), but was most common among accident victims (*n* = 4). The blood alcohol concentration (BAC) ranged from 0.23 to 3.4 g/L in the whole study material with the highest concentrations found among the accident victims. Toxicology was not tested in nine cases; all died in hospital, mostly due to burns.

**Table 2.** Toxicology findings in explosion-related deaths in Sweden, 2000–2018.

Toxicology (*n* = number of fatalities)	Accidents (*n* = 54)	Suicides (*n* = 18)	Homicides (*n* = 6)	Undetermined MOD (*n* = 9)	Total
Toxicology tests positive, *n*/total (%)	24/54 (44%)	13/18 (72%)	4/6 (67%)	8/9 (89%)	49/87 (56%)
Toxicology tests negative, *n*/total (%)	22/54 (41%)	5/18 (28%)	1/6 (17%)	1/9 (11%)	29/87 (33%)
Toxicology not tested, *n*/total (%)	8/54 (15%)	0/18 (0%)	1/6 (17%)	0/9 (0%)	9/87 (10%)
Licit drug detected, *n*/total of all tested (%)	10/46 (22%)	8/18 (44%)	1/5 (20%)	3/9 (33%)	22/78 (28%)
Benzodiazepines, *n*/total licit drug detected (%)	1/10 (10%)	2/8 (25%)	0/1 (0%)	1/3 (33%)	4/78 (5%)
Antidepressants, *n*/total licit drug detected (%)	1/10 (10%)	1/8 (13%)	0/1 (0%)	0/3 (0%)	2/78 (3%)
Neuroleptics, *n*/total licit drug detected (%)	0/10 (0%)	2/8 (25%)	0/1 (0%)	0/3 (0%)	2/78 (3%)
Opiates, *n*/total licit drug detected (%)	2/10 (20%)	4/8 (50%)	0/1 (0%)	2/3 (67%)	8/78 (10%)
Other licit drugs,^a^ *n*/total licit drug detected (%)	9/10 (90%)	7/8 (88%)	1/1 (100%)	0/3 (0%)	17/78 (22%)
Benzene/heptane/hexane/toluene detected, *n*/total of all tested (%)	2/46 (4%)	2/18 (11%)	1/5 (20%)	6/9 (67%)	11/78 (14%)
COHb detected, *n*/total of all tested (%)	9/46 (20%)	9/18 (50%)	0/5 (0%)	8/9 (89%)	26/78 (33%)
COHb concentration % in blood – range	2–50	2–63	–	4–75	2–75
Cyanide detected, *n*/total of all tested (%)	6/46 (13%)	1/18 (6%)	0/5 (0%)	3/9 (33%)	10/78 (13%)
Cyanide concentration, μg/g blood – range	0.2–1.4	0.13	–	0.11–3.4	0.11–3.4
Alcohol^b^ detected, *n*/total of all tested (%)	10/46 (22%)	5/18 (28%)	0/5 (0%)	0/9 (0%)	15/78 (19%)
BAC (g/L)					
0.2 to <1	4	2	0	0	6
1–3.4	6	0	0	0	6
Mean BAC among test positive	1.56	1.14	0	0	1.49
BAC <0.2 g/L and alcohol concentration in urine >0.2	0	3	0	0	3
Alcohol^b^ test negative, *n*/total of all tested (%)	34/46 (74%)	13/18 (72%)	5/5 (100%)	9/9 (100%)	61/78 (78%)
Alcohol^b^ not tested, *n*/total of all toxicology tested (%)	2/46 (4%)	0/18 (0%)	0/5 (0%)	0/9 (0%)	2/78 (3%)

^a^ a.o. antihistamines, paracetamol, metoprolol, amiodarone and anaesthetics.

^b^ Alcohol = ethanol.

MOD: manner of death; COHb: carboxyhaemoglobin; BAC: blood alcohol concentration.

The most frequent toxicological findings among accidental fatalities were alcohol and licit drugs, followed by carboxyhaemoglobin (COHb) and cyanide (Table 2). Among alcohol test-positive cases, the majority had a BAC ≥1 g/L. Non-psychotropic drugs were more common than psychotropic drugs. All victims of occupational accidents tested negative for alcohol and illicit drugs.

In suicides, COHb was the most common toxicological finding, followed by licit drugs and alcohol. Psychotropic drugs and opiates predominated among licit drugs. There was no significant difference between the share of alcohol positive and alcohol negative victims in accidental deaths versus suicides *(χ*^2^ = 0.178, df = 1, p = 0.673).

Gas intoxication was common (44%) in victims of undetermined MOD, and one-third were positive for benzodiazepines and opiates (Table 2).

## Discussion

Explosion mechanisms and categories of the MOD found in a civilian setting in peacetime differ from those found in a combat setting, thus implicating different epidemiological aspects of such deaths. However, previous research from a non-combat context has been limited. The principal findings of the present study were a low annual incidence of blast deaths in a civilian setting, a variety of injury mechanisms across all MODs, and a predominance of accidental fatalities.

The present study confirms the variation of circumstances and injuries and low fatality incidence found in a few previous studies.^11,12,14^ The annual incidence of explosion-related deaths in Sweden in the 1980s (12/10 million population)^11^ has now decreased to 4.9 annual deaths per 10 million population. Technology improvements, preventive measures in the occupational environment and suicide prevention may possibly explain this development. The decline in incidence in more recent years, from 2000 to 2018, was indicative but not statistically significant.

According to official statistics (available for 2001–2018),^15^ the number of people treated in hospital for accidental blast injuries, and the number of accidental blast fatalities found in the present study, constitute the ratio of 1382:52, which is similar to the ratio found in a Finnish study.^12^ In congruence with the World Health Organisation’s (WHO's) injury pyramid,^16^ the fraction of non-lethal injuries in the pyramid basis is much larger than the fraction of deaths on the top of the pyramid. Yet, the number of all explosions not causing any injury is not known, as there is no national surveillance of all such incidents in Sweden. However, an increase of grenade attacks in three major Swedish cities in 2011–2016 has been reported.^17^ The study found only one death and nine injured persons during the study period, but the number of incidents of detonated hand grenades was much higher (*n* = 77).^17^ The observed increase of grenade attacks was suggested to reflect a change in the violence pattern in urban areas, most likely related to criminal groups.^17^

Recently, some legislative changes have been introduced in Sweden, e.g. a stricter law and penalties regarding violation in the handling of explosives.^18^ In addition, an amnesty of explosives during 2018 resulted in reduced availability of explosives in the society. However, most explosives handed over to the police during this amnesty did not come from criminals but from forestry and farming businesses.^19^ Restricting availability of explosives to criminals is a challenging task. In addition, a new and stricter regulation for fireworks use in Sweden was introduced in 2019,^20^ which will hopefully contribute to the prevention of firework-related accidental injuries and deaths.

The majority of the victims across all manners of death were males,^11,12,14^ corresponding to the WHO reporting of males being more at risk of death from injuries and violence in general. Males are also more likely to take their own life than females^21^ and are also more prone to use a violent suicide method.^22^ As previous epidemiological studies are rare and due to the heterogeneity of the injury panorama, the age patterns are difficult to relate to previous research. However, overall suicide rates increase with age,^21^ thus possibly explaining a higher mean age of suicide victims than of victims of other MODs in explosion fatalities. Similarly to explosion deaths, also other violent deaths, such as firearm deaths, exhibit a male predominance and an age pattern with the oldest victims in suicides and the youngest in homicides.^23^

As expected, accidental deaths predominate over suicides and homicides in Sweden and elsewhere.^11,12,14^ Occupational accidental deaths were mostly related to the work environment with explosives and none of these victims tested positive for alcohol or illicit drugs. Work-related accidental deaths accounted for approximately one-third of all accidental fatalities, as in a previous Swedish study,^11^ but this figure was lower than in an Australian study.^14^ Different study designs, settings and environmental factors could possibly explain this pattern variability between studies.

Unexpectedly, the present study found four preteen deaths, while a previous Swedish study presented only one accidental child death,^11^ and no child mortality was reported in an Australian study.^14^ In addition, two of the minors accounted for one-third of all homicide victims in the present study.

As expected, the number of deaths at the scene was high and also higher among intentional than among unintentional deaths. The pattern of suicides did not change since the 1980s,^11^ still with two-thirds of cases occurring outdoors. Explosion as a suicide method is rare, hence why all cases need to be meticulously investigated for the possibility of other MODs, especially homicide.^24^

The pathophysiology of blast injuries may manifest as a primary, secondary, tertiary and quaternary injuries.^25–27^ Primary blast injury is related to the blast wave in high-order explosions causing barotrauma to the lungs, ear, brain or abdominal organs. Debris may penetrate the body surface causing secondary blast injuries. If the blast wind relocates a victim or causes a building to collapse, tertiary blast injuries (penetrating or blunt force trauma) may occur. An explosion may also include fire, radiation, smoke or toxins, and exposure to such factors leads to quaternary blast injuries, such as burns and intoxication.^25–27^

Most injuries in the present study were secondary and quaternary. Fire is commonly related to explosions, hence findings of severe burns and toxic gas inhalations were expected. Primary blast injuries are less common and more indistinct^28^ and did not cause any deaths in the present study. Besides the primary blast injury, some potentially lethal injuries to e.g. blood vessels, may hide behind visually apparent injuries and lead to a missed diagnosis.^29^

In addition, reviewing health risks of explosions in a civilian setting showed that both physical and psychological long-term effects may follow a blast injury.^30^ Adequate efforts early after an explosion may benefit the final outcome of the injury.^30^

Although the majority of cases in the present study were not alcohol and illicit drugs positive, positive toxicological findings have been observed in victims of intentional deaths and non-occupational accidents. The additional contribution of opiates and psychotropic drugs found in suicides is indicative of risk factors, risk behaviours and mental health issues.

Injury-related deaths may be prevented by a variety of strategies.^16^ Some measures may be common for all MODs in blast injury, e.g. targeting the availability of explosives and other measures aiming at specific risk factors, such as in suicides. Yet, the issue of blast injury prevention may be challenging in a civilian setting due to the variety of circumstances and injury mechanisms. The Haddon matrix^31^ has been used for identifying and mapping factors involved in different stages of incidents causing injury (e.g. traffic crashes). Injury preventing actions are necessary for the pre-event, event and post-event phases and may be differentiated at the agent, host and environmental level. The matrix has been applied in the review of mass casualty incidents in the underground mining industry^32^ and for suggesting medical strategies in a terrorist bombing scenario.^33^ In the pre-event phase, restriction of the availability of explosives, the host safety preparedness and environmental factors of providing availability of the emergency response are some important actions.^33^ Furthermore, control of the secondary damage, prompt medical response in the event phase and post-event health care for injured persons represent some of the relevant factors adjustable by adequate measures.^33^ Thus, the Haddon matrix represents a useful tool also regarding blast injury prevention work and research.

The use of longitudinal and nationwide register data represents a strength of this study. There should be no out-of register cases since all blast fatalities should have undergone a medico-legal autopsy according to Swedish law and regulations. The data are thus representative of all explosion-related deaths in Sweden during the study period. Furthermore, all cases of undetermined MOD were re-evaluated based on the register data, avoiding individual differences between forensic pathologists in the classification of such cases. The study limitations are related to its retrospective design, missing data about place of death in six accidental deaths, and missing toxicology data for nine cases that were not tested. Furthermore, results regarding intentional deaths need to be interpreted with caution due to the small number of cases.

## Conclusions

The incidence of explosion fatalities in Sweden has decreased since the 1980s. This decrease seems, however, to have slowed down in the last decade. Unintentional deaths predominated with a variety of explosion mechanisms. Findings of blast deaths in children and involvement of explosives in homicides, suicides and occupational accidental deaths should initiate actions targeting safety in the working environment and restricting the availability of explosives in both legal and illegal activities.

A survey of epidemiological data concerning blast deaths offers valuable insights into causes and risks related to these fatalities. Further monitoring could be helpful for the evaluation of the possible impact of recent legislation changes in Sweden. Nevertheless, surveillance and studies also of non-lethal blast injuries are needed. The rarity of blast deaths and the characteristics of multiple and different types of blast injuries may be challenging in prevention efforts.
